# How Well Can we Diagnose Autism in Adults? Evaluating an Informant-based Interview: The Dutch Developmental, Dimensional and Diagnostic Interview – Adult Version (3Di-Adult)

**DOI:** 10.1007/s10803-023-06069-5

**Published:** 2023-08-02

**Authors:** L.J.G. Krijnen, K. Greaves-Lord, W. Mandy, K.J.S. Mataw, P. Hartog, S. Begeer

**Affiliations:** 1 Child and Adolescent Studies, Utrecht University, Utrecht, The Netherlands; 2 Department of Clinical, Neuro and Developmental Psychology, Vrije Universiteit Amsterdam, Amsterdam Public Health Research Institute, Amsterdam, The Netherlands; 3 Department of Clinical Psychology and Experimental Psychopathology, University of Groningen, Groningen, The Netherlands; 4 Autism Team Northern-Netherlands, Jonx, Department of (Youth) Mental Health and Autism of Lentis Psychiatric Institute, Groningen, The Netherlands; 5 Research Department of Clinical, Educational and Health Psychology, UCL, London, UK

**Keywords:** Autism, Diagnostic interview, Adults, Psychometric properties, Informant-report

## Abstract

The current study evaluated a brief, informant-based autism interview: the Developmental, Dimensional and Diagnostic Interview – Adult Version (3Di-Adult). Feasibility, reliability and validity of the Dutch 3Di-Adult was tested amongst autistic participants (n = 62) and a non-autistic comparison group (n = 30) in the Netherlands. The 3Di-Adult consists of two scales based on DSM-5 criteria: A scale ‘Social communication and social interaction’ and B scale ‘Restricted, repetitive patterns of behavior, interests or activities’. ROC curves were used to determine cut-off scores for the A and the B scale, using an ASD diagnosis made by an independent clinician as the criterion. Mean administration time was 42 min. Internal consistency of the A scale (α = 0.92) and the B scale (α = 0.85) were good. Inter-rater reliability (ICCs = 0.99) and inter-rater agreement (ICCs ≥ 0.90) were promising. The 3Di-Adult showed good sensitivity (80.6%) and specificity (93.3%). Positive and negative predictive value were 96.2% and 70.0% respectively. Comparisons with the Autism-Spectrum Quotient-Short to investigate the convergent validity showed moderate, significant correlations with the 3Di-Adult in the total sample. Males, as compared to females, displayed significantly more autistic features on the 3Di-Adult. No relationship was found of the 3Di-Adult with education level, intelligence and age of the participants or informants. The feasibility and psychometric properties of the Dutch 3Di-Adult are promising, indicating that it can be a time-efficient, valid and reliable tool to use in diagnosing autism in adults according to DSM-5 criteria.

Autism Spectrum Disorder (ASD, from here on autism or ASD) is a neurodevelopmental condition characterized by persistent deficits in social communication and social interaction and restricted, repetitive patterns of behavior, interests, or activities (American Psychiatric Association [APA], 2013). Although autism can already be diagnosed at the age of two, there is a significant number of people who do not get diagnosed way into adulthood (Lai & Baron-Cohen, 2015; Steiner et al., 2012). Diagnostic instruments to assess autism are often developed for children, resulting in a scarcity of validated diagnostic tools for adults (Howlin & Moss, 2012; National Institute for Health and Care Excellence, 2016). Undiagnosed adults are at high risk for experiencing emotional and functional impairments as a result of their unrecognized autistic symptoms (Lai & Baron-Cohen, 2015). Therefore, more research regarding methods to assess autism in adults is needed. The current study investigates the psychometric properties of the Dutch version of a relatively new informant-based interview for diagnosing adults with autism: the Developmental, Dimensional and Diagnostic Interview – Adult Version (3Di-Adult; Mandy et al., 2018).

There are various reasons why autism may go unnoticed until adulthood. People with a normal to high intelligence, more subtle autistic features, a strong support-system or well-acquired camouflaging techniques, generally display less explicit autistic symptoms, causing these symptoms to be missed in the diagnostic process (Bargiela et al., 2016; Cook et al., 2021; Lai et al., 2011). Furthermore, core symptoms of autism appear to become less apparent as the person ages (e.g. due to acquired camouflaging techniques, training of skills, environmental adjustments), making it more difficult to diagnose autism in adults (Charman et al., 2011; Shattuck et al., 2007). Another problem concerns the finding that diagnostic tools developed for children are not always appropriate or sensitive enough to be used in adults (Fusar-Poli et al., 2017). Furthermore, ASD seems to be more difficult to diagnose in females (Young et al., 2018). Therefore, an instrument specifically developed to detect autism in adults (as defined by the DSM-5 and ICD-11) is of high importance.

The British National Institute for Health and Care Excellence (NICE) acknowledged the problems in diagnosing adults with autism and developed a clinical guideline regarding this matter (National Institute for Health and Care Excellence, 2016). The diagnostic procedure usually consists of self-report measures on current behaviors or feelings (e.g. Autism-Spectrum Quotient; AQ; Baron-Cohen et al., 2001), observational assessments of real-time behavior (e.g. Autism Diagnostic Observation Schedule; ADOS-2; Lord et al., 2000) and informant-based assessments in which a close other (e.g. parent, sibling) reports about the current and early childhood behaviors. Well-known informant-based instruments are the Autism Diagnostic Interview-Revised (ADI-R; Lord et al., 1994) or the Diagnostic Interview for Social and Communication Disorders (DISCO; Wing et al., 2002). However, known disadvantages of these measures are their high costs and long administration time (ADI-R: 1.5 h; DISCO up to 3 h). Furthermore, the ADI-R may be less reliable in adults without an intellectual disability, as the sensitivity was found to be low (i.e. 55%) (Fusar-Poli et al., 2017). Although sensitivity of the DISCO was found to be good, specificity could not be properly evaluated because there was no adult control group (Kent et al., 2013).

Given the importance of integrating information on early development in the diagnostic work-up, informant-based developmental interviews play a pivotal role in diagnosing adults with autism. Therefore, an informant-based instrument that is time-efficient, valid and reliable in diagnosing adults with autism is of great interest. Other major advantages of informant-based interviews are that the everyday natural behavior of the person can be assessed, that cannot be evoked in an observational setting. Moreover, interviews can provide broader information about the person, including the strengths and challenges that are encountered in daily life, which is useful for further guidance.

A few years ago, the Developmental, Dimensional and Diagnostic Interview – Adult Version (3Di-Adult; Mandy et al., 2018) has been developed, based on the initial child version which has been studied and compared to existing instruments (Evers et al., 2020, 2021; Slappendel et al., 2016). This informant-based interview assesses development and current functioning of the person, using DSM-5 criteria for autism. Preliminary results showed that the 3Di-Adult was fast, reliable, valid, and easy to assess, since it can be administered over the telephone in less than 40 min (Mandy et al., 2018). This enables the clinician to collect information regarding both the early development and current functioning of the person, without the expensive and time-consuming processes entailed by the ADI-R and DISCO. The 3Di-Adult can be used to establish a diagnostic classification, but also for mapping the profile of autistic features of the individual, which is useful for further guidance (American Psychiatric Association [APA], 2013). The 3Di-Adult was preliminary tested in England, comparing three groups with a mean age ranging from 28 to 32: ASD participants (*n* = 39), control participants (*n* = 29) and clinical control participants (*n* = 20). Results showed excellent specificity (92%) and sensitivity (95%). Internal consistency was high, and inter-rater agreement was very high. These promising results encouraged further development and investigation of the 3Di-Adult, by creating and testing a Dutch version of this interview to evaluate whether this instrument is also a valuable tool in the Netherlands.

The current study aimed to investigate the feasibility and psychometric properties – i.e. reliability and validity - of the Dutch version of the 3Di-Adult, in a sample of adults with and without an ASD diagnosis. This information will provide us with more information on whether the Dutch 3Di-Adult can be used in clinical practice as well as for research.

## Methods

### Participants

All ASD participants and part of the control participants were recruited via the ‘Nederlands Autisme Register’ (NAR, for more information: https://nar.vu.nl/), which is a Dutch database of data from people with ASD and controls without ASD, which has been approved by the Permanent Committee on Science and Ethics (VCWE) of the VU University Amsterdam (number: VCWE-2020-041R1). This study was part of a larger study for which ASD participants were recruited via the NAR. Permission to contact one of their relatives was asked. Control participants were recruited for this study only, partly through the NAR, but also through personal networks and online recruitment. If control participants were not registered at the NAR yet, they were asked to register, so that more information about them (demographics, other autism measurements) could be obtained. The only exclusion criterion for the control group was an ASD diagnosis. Inclusion criteria for both groups were (1) age of 18 years or older, and (2) permission to contact one of their relatives to assess the 3Di-Adult. The informant, which refers to the person with whom the interview was conducted, was required to have sufficient knowledge of the Dutch language and had to be sufficiently involved in the participant’s (early) life. Therefore, a parent or sibling was the preferred person to conduct the interview with. For the ASD group, participants had to have obtained their ASD diagnosis from either a psychologist, remedial educationalist, psychiatrist, doctor or a multidisciplinary team. Participants’ characteristics and autistic features other than 3Di-Adult information were obtained by the standardized questionnaires that were filled out after registering at the NAR. See Table 1 for the participants’ characteristics.

**Table 1:** Sample characteristics (n = 92)

	ASD group (*n* = 62)		Control group (*n* = 30)		Group difference	
	*Informant*	*Participant*	*Informant*	*Participant*	*Informant*	*Participant*
Gender^a^					ns	ns
Male, *n* (%) Female, *n* (%)	9 (14.8%) 52 (85.2%)	25 (40.3%) 36 (58.1%)	7 (23.3%) 23 (76.7%)	6 (21.4%) 22 (78.6%)		
Relationship					ns	-
Mother Father Sibling Partner Other^b^	42 (67.7 %) 4 (6.5%) 9 (14.5%) 4 (6.5%) 3 (4.8%)	- - - - -	17 (56.7%) 1 (3.3%) 6 (20.0%) 6 (20.0%) 0 (0.0%)	- - - - -		
Age, *in years*^c^					ns	ns
Range Mean (SD)	28-81 61.56 (12.58)	20–54 41.53 (9.00)	37-82 62.40 (9.25)	21-74 44.96 (17.67)		
Intelligence level^d^					-	ns
Low Medium High	- - -	9 (14.8%) 10 (16.4%) 42 (68.9%)	- - -	0 (0.0%) 5 (17.9%) 23 (57.1%)	- - -	
Education level^e^					*p* <.01	p < .05
Low, *n* (%) Medium, *n* (%) High, *n* (%)	17 (27.9%) 18 (29.5%) 26 (42.6%)	10 (16.1%) 18 (29.0%) 32 (51.6%)	3 (10.3%) 3 (10.3%) 23 (79.3%)	0 (0.0%) 4 (13.3%) 18 (60.0%)		
Ethnicity^f^					-	ns
Dutch	-	60 (96.8%)	-	27 (96.4%)		
Other^g^	-	2 (3.2%)	-	1 (3.6%)		
Age of diagnosis^h^						
Range Mean	- -	5.00-50.92 33.35 (11.30)	- -	- -	- -	- -

*Note*. ASD = Autism Spectrum Disorder; ns = not significant on an alpha level of 0.05 using an independent samples t-test for continuous data and either a Chi squared test or Fisher Exact test for categorical data

^a^ For two control participants and one ASD participant, self-reported gender was unknown

^b^ ‘Other’ referred in two cases to a friend and one case to a son

^c^ For two control participants, age was unknown

^d^ Low = IQ < 86, medium = 86–115, high > 115. Intelligence level is based on either self-reported intelligence estimates, or results from an IQ test. Two control participants’ intelligence level was missing. IQ estimates for informants are unknown

^e^ Education level is classified as prescribed by the ‘Standaard Onderwijsindeling’ (Centraal Bureau voor de Statistiek, 2021). Information was missing for one control informant and one ASD informant. For the participants, information was missing for 8 control participants and 2 ASD participants

^f^ No information regarding the ethnicity of the informants. For 2 control participants, information regarding ethnicity was missing

^g^ One ASD and one control participant that were part of the ‘Dutch’ category also had an ‘other’ nationality besides their Dutch nationality

^h^ Data of four ASD participants were missing

### Materials

**Developmental, Dimensional and Diagnostic Interview – Adult Version** (3Di-Adult; Mandy et al., 2018). The 3Di was originally developed to assess ASD in children and adolescents (Skuse et al., 2004). Psychometric properties have shown to be strong, with high levels of inter-rater reliability and test-retest agreement (intraclass correlation coefficients > 0.86). Criterion validity, in which the ADI-R formed the comparison, showed to be excellent and sensitivity and specificity were high (1.0; >0.97, respectively). The Dutch version of the 3Di child version showed sufficient psychometric properties as well (Slappendel et al., 2016).

Mandy and colleagues (2018) developed an adult version based on the original 3Di, which led to the 3Di-Adult – a structured interview. They selected an initial pool of items that had already shown to be especially discriminating (Chuthapisith et al., 2012; Santosh et al., 2009; Skuse et al., 2004). Hence, the items were distributed across the DSM-5 criteria, resulting in two scales: the A scale ‘social communication and social interaction’ and the B scale ‘restricted, repetitive patterns of behavior, interests or activities’. Both scales consist of subscales that directly map onto the DSM-5 criteria – i.e. A scale: A1 ‘social emotional reciprocity’ (12 items), A2 ‘nonverbal behavior for social interaction’ (17 items), and A3 ‘forming, maintaining and understanding relationships’ (18 items) and B scale: B1 ‘stereotyped, repetitive behavior and language’ (3 items), B2 ‘insistence on sameness’ (5 items), B3 ‘restricted fixated interests’ (5 items), and B4 ‘abnormal sensory response’ (5 items). The total interview contains 65 scored items of which 47 belong to the A scale and 18 items to the B scale. In addition, there are 4 items regarding the age of reaching developmental milestones (i.e. sitting unsupported, first steps, first words, first sentence), which are not included in the DSM-5 algorithm to calculate the final scores, but give additional clinical information that is relevant for classifying ASD (Greaves-Lord et al., 2022). The majority of the items (i.e. 48) assess current behavior, and the remaining items (i.e. 21) assess (early) childhood behavior.

Items are scored on either a 3-point Likert scale (0 = often, 1 = sometimes, 2 = never), or a 4-point Likert scale (0 = No, 1 = Yes, minimal, 2 = Yes, persistent, 3 = Yes, persistent with functional impairment). All items with a score of 3 were recoded to 2 ensuring an equivalent weighting of every item. Items to which participants did not know the answer, were recoded to 0. Fifteen items are reversely phrased and need to be recoded, so that higher scores reflect more autistic symptoms. The subscales are calculated by averaging the scores of the corresponding items, resulting in a maximum score of 2 for every subscale. The A and B scales were calculated by summing the averaged scores of the subscales, resulting in a maximum score of 6 for the A scale (sum of 3 subscales) and 8 for the B scale (sum of 4 subscales). A score above the cut-off on the A scale *and* the B scale is indicative for an ASD diagnosis. Mandy and colleagues (2018) calculated cut-off scores for the A scale and the B scale for the English version of the 3Di-Adult, based on their sample using ROC curves. The current study also used ROC curves to calculate cut-off scores for the A and the B scale of the Dutch 3-Di Adult, based on our sample of Dutch participants.

In the current study, the 3Di-Adult was translated to Dutch by two independent researchers that were native in Dutch and fluent in English, taking into consideration the Dutch translation of the 3Di child version (Slappendel et al., 2016). Consensus was made about the final translation, in cooperation with a third independent researcher with knowledge about the purpose of the 3Di. Then, this version was back translated by a native English and Dutch speaker. Based on this back translation, adjustments were made to the Dutch translation. Four pilot assessments took place in which the interview was tested. Hence, the final version was made.

**Autism-Spectrum Quotient-Short** (AQ-Short; Hoekstra et al., 2011). The Dutch, abridged adult version of the original 50-item questionnaire was used to assess autistic traits. This self-report measure consists of 28 items, divided over two higher-order factors: ‘social behavioral difficulties’ (23 items e.g. “I enjoy social occasions”) and ‘a fascination for numbers and patterns’ (5 items e.g. “I am fascinated by dates”). Items are rated on a 4-point Likert scale, ranging from 1 = ‘definitely agree’ to 4 = ‘definitely not agree’. Total scores could range from 28 to 112, with higher scores representing more autistic traits. A cut-off score of 65 has been suggested for screening purposes (Hoekstra et al., 2011). Psychometric properties have shown to be good, with high sensitivity and specificity (Hoekstra et al., 2011).

In the current study, all participants filled out the AQ-Short when registering at the NAR. All ASD participants obtained a total AQ-Short score above 65 (mean = 87.00, SD = 8.91). Except for one control participant who scored 66, all control participants scored below the cut-off of 65 (mean = 47.36, SD = 9.07).

### Procedure

Participants with ASD were recruited for a larger study, to which this study is linked. Permission was asked to contact an informant of their inner circle (e.g. parents/sibling) via e-mail. Participants were free to choose their informant, but a parent or sibling was recommended by the researchers, given their involvement during early development. Once participants gave permission, the informant was called to ask whether he/she was willing to participate. When the informant agreed to take part in the study, an information letter including an informed consent was sent by post, and the informant was called to make an appointment to administer the 3Di-Adult. The procedure for control participants was slightly different, since these participants were not part of the larger study. Control participants were also approached per e-mail to ask permission for contacting one of their close relatives. The informants then received an e-mail to ask for permission, with an information letter attached to the e-mail. If they replied that they were willing to participate, one of the researchers called them to make an appointment to administer the interview. No incentives were provided to the participants and the informants.

The 3Di-Adult interview was administered over the phone by trained interviewers. Interviewers consisted of master students and researchers who received a training by a licensed professional. At the end of the training, all trainees had to code one golden standard interview to test their reliability. All interviewers produced a reliability rating of at least 85%.

In order to assess the inter-rater reliability and the inter-rater agreement - see the analyses section for an explanation regarding these concepts - audio recordings from three random participants were made. Consent to record the interview was asked before the start of the interview. After the first researcher finished the interview, two other researchers rated the interviews while being unaware of the condition (i.e. ASD or control participant) and the scoring of the previous rater.

### Analyses

IBM SPSS Statistics version 25 was used to analyze the data. First, the number of missing data and the administration time of the 3Di-Adult was described. If no more than 50% of the 3Di-Adult items were missing, scores for the A and B (sub)scales were calculated. Secondly, differences between the ASD and control group on 3Di-Adult (sub)scales were tested using independent samples t-test. Effect sizes were calculated, using Glass Delta when variances between the groups were unequal. If variances were equal, Hedges’ *g* was reported which is suitable for unequal sample sizes between the groups. Effect sizes of 0.2, 0.5 and 0.8 were considered small, medium and large respectively (Cohen, 1988). To check whether the A and B scale of the 3Di-Adult measure the same underlying construct, i.e. ASD, correlations between the A and B scales were calculated. Spearman’s Rho (*r*_s_) was used to protect from outliers and non-normal distributed data. The strength of the correlations were interpreted as follows: 0.00 to 0.30 was considered weak, 0.40 to 0.60 as moderate and ≥ 0.70 as strong (Akoglu, 2018).

Hence, reliability - i.e. internal consistency, inter-rater agreement and inter-rater reliability - was assessed. Cronbach’s alpha was calculated to assess internal consistency of the 3Di-Adult scales. The inter-rater reliability (IRR) and the inter-rater agreement (IRA) were calculated using the intraclass correlation coefficient (ICC). IRR and IRA are terms that are often used interchangeably, but there is a technical distinction: IRR can be defined as the extent to which raters consistently distinguish between different items on a measurement scale, whereas IRA relates to the extent to which different raters assign the exact same rating to the same item (Gisev et al., 2013). To calculate the IRR and IRA, the ICC estimates and their 95% confidence interval were based on a mean-rating of the three raters (*k* = 3), consistency, two-way random model. IRR and IRA were calculated per A and B scale. To interpret the ICC, guidelines of Koo and Li (2016) were used, stating that the 95% confidence interval of the ICC should be interpreted according to the following guidelines: <0.5 is poor, 0.5 to 0.75 moderate, 0.75 to 0.90 good and ≥ 0.90 excellent.

To investigate criterion validity of the 3Di-Adult, the 3Di-Adult results were compared to a gold standard, i.e. the ASD diagnosis made by an independent clinician. Cut-off scores of the 3Di-Adult were determined for the A and the B scale separately, using ROC curves in which the clinical diagnosis formed the criterion measure. Area Under Curves (AUC) were estimated with values ranging from 0.50 to 0.70 referring to low accuracy, 0.70 to 0.90 to moderate accuracy and 0.90 to 1.00 to high accuracy (Fischer et al., 2003). In order to determine cut-off scores for the A and B scale, Youdens *J* was used. The highest Youdens *J* index was selected for the A and the B scale, which refers to the optimal trade-off between sensitivity and specificity. A score above the A scale and the B scale is considered to be indicative for ASD. Participants were then assigned to one of the four following groups: (1) true positives (i.e. score above the cut-off for ASD on the A *and* B scale of the 3Di-Adult + clinical ASD diagnosis), (2) true negatives (i.e. score below the cut-off for ASD on 3Di-Adult A and/or B scale + no clinical ASD diagnosis), (3) False negatives (i.e. score below the cut-off for ASD on 3Di-Adult A and/or B scale + clinical ASD diagnosis), (4) False positives (i.e. score above the cut-off for ASD on the A *and* B scale of 3Di-Adult + no clinical ASD diagnosis). Hence, specificity, sensitivity, positive predictive value (PPV) and negative predictive value (NPV) of the 3Di-Adult were calculated. Sensitivity and specificity values above 70% were considered good (Glascoe, 2000). No general cut-off scores for a good PPV/NPV exist as these values are dependent on the prevalence of control versus ASD participants in the sample (Tenny & Hoffman, 2020).

Construct validity was assessed by examining the convergent and divergent validity. Convergent validity was tested by investigating the correlations between the two higher-order scales of the AQ-Short (i.e. ‘social behavioral difficulties’ and ‘a fascination for numbers and patterns’) to the A and B scale of the 3Di-Adult respectively, using Spearman’s Rho. The first AQ-Short scale, ‘social and behavioral difficulties’, was expected to correlate significantly to the 3Di-Adult A scale as both scales seem to be conceptually close. The AQ-Short scale ‘a fascination for numbers and patterns’ on the other hand was expected to be significantly correlated to the 3Di-Adult B scale as these scales seem to measure comparable constructs.

Divergent validity was assessed by exploring relationships between the 3Di-Adult A and B scores and participants’ and informants’ characteristics, i.e. gender, age, education level (3 levels as prescribed by the ‘Standaard Onderwijsindeling’; Centraal Bureau voor de Statistiek, 2021) and IQ levels (3 levels, i.e. low = IQ < 86, medium = 86–115, high > 115 as assessed by an intelligence test when available, if not, a self-reported estimation of one’s intelligence level was used). Independent samples t-test, Spearman’s Rho and 2 ANOVA tests were conducted, respectively.

## Results

### Administration Time and Missing Data

The mean administration time was 41.78 min, ranging from 19 to 84 min. Interviews in the ASD group took significantly longer (mean = 46.55 min, *SD* = 13.46, ranging from 23 to 84 min) than in the control group (mean= 31.93, *SD* = 10.58 min ranging from 19 to 60 min), *p* < .001.

Missing data ranged from 0 to 30 out of 69 items, with an average number of missing items of 5.80 (8.40%), and a median of 3 missing items. Of the 47 scored items of the A scale, on average there were 3.63 missing items (7.72%), with a median of 2 missing items. For the B scale, consisting of 18 scored items, on average answers on 1.36 items were missing (7.56%) with a median of 0. For every participant, no more than 50% of the items were missing, enabling us to calculate scores on the 3Di-Adult for all participants.

No significant correlation was found between the number of missing items on the A and the B scale and the administration time (*r*_*s*_ < − 0.12, *p’s* > 0.25). The number of missing items on the A and the B scale did not significantly differ between the ASD and control group (*p’s > .*54).

### 3Di-Adult: A and B Scale Scores Per Group

Table 2 shows the scores on the 3Di-Adult, showing that the ASD participants scored significantly higher than the control participants on all the (sub)scales (*p*’s < 0.001). All effect sizes indicated large differences, except for the B1 subscale which showed a moderate effect size.

**Table 2:** *Scores on each (sub)scale of the 3Di-Adult, per group*

	ASD (*n* = 62)	Controls (*n* = 30)	Difference between groups	Effect size of group difference
A scale ‘Social communication and social interaction’				
Mean (*SD*) Range	2.04 (0.89) 0.06-3.64	0.50 (0.51) 0.00-1.92	*p* < .001	Glass’s delta = -1.74
A1 subscale: ‘Social emotional reciprocity’				
Mean (*SD*) Range	0.63 (0.37) 0.00-1.58	0.16 (0.22) 0.00-0.83	*p* < .001	Glass’s delta = -1.27
A2 subscale: ‘Nonverbal behavior for social interaction’				
Mean (*SD*) Range	0.65 (0.36) 0.00-1.29	0.15 (0.18) 0.00-0.71	*p* < .001	Glass’s delta = -1.40
A3 subscale: ‘Forming, maintaining and understanding relationships’				
Mean (*SD*) Range	0.77 (0.35) 0.068-1.56	0.20 (0.19) 0.00-0.67	*p* < .001	Glass’s delta = -1.65
B scale: ‘Restricted, repetitive patterns of behavior, interests or activities’				
Mean (*SD*) Range	3.42 (1.34) 0.20–6.67	0.73 (0.81) 0.00-2.60	*p* < .001	Glass’s delta *=* -2.00
B1 subscale: ‘Stereotyped, repetitive behavior and language’				
Mean (*SD*) Range	0.41 (0.47) 0.00–2.00	0.13 (0.19) 0.00-0.60	*p* < .001	Glass’s delta = -0.62
B2 subscale: ‘Insistence on sameness’				
Mean (*SD*) Range	1.33 (0.59) 0.00–2.00	0.19 (0.29) 0.00–1.00	*p* < .001	Glass’s delta = -1.94
B3 subscale: ‘Restricted fixated interests’				
Mean (*SD*) Range	0.95 (0.46) 0.00-1.80	0.25 (0.32) 0.00–1.00	*p* < .001	Hedges’ *g* = -1.67
B4 subscale: ‘Abnormal sensory response’				
Mean (*SD*) Range	0.72 (0.44) 0.00-1.80	0.17 (0.20) 0.00-0.80	*p* < .001	Glass’s delta = -1.25

*Note.* ASD = Autism Spectrum Disorder; *SD* = Standard Deviation. Differences between the ASD and control group were tested using independent samples t-tests

For the total sample, Spearman’s Rho indicated a significant, strong positive relationship between the A and B scale, *r*_*s*_ = 0.77, *p* < .001, indicating that both scales measure the same underlying construct. Within the ASD group, a significant correlation of moderate strength was found between the A and B scale, *r*_*s*_ = 0.46, *p <* .001, and a significant strong positive correlation was found between the scales for the control group, *r*_*s*_ = 0.80, *p* < .001.

### Reliability

#### Internal Consistency

Internal consistency of the A scale was excellent (α = 0.92) and internal consistency of the B scale was good (α = 0.85).

#### Inter-rater Reliability and Inter-rater Agreement

According to the ICC estimates, the IRR for scale A was excellent, ICC (2,3) = 0.99, 95%CI [0.90 to 1.00], *F*(2, 4) = 110.34, *p* < .001. The IRR for scale B was good to excellent, ICC (2,3) = 0.99, 95%CI [0.84 to 1.00], *F*(2,4) = 65.60, *p* < .01. This indicates that all 3 raters discriminated between the different items in a consistent way.

The IRA for the A scale was difficult to interpret, because of the broad 95% confidence interval, which is probably due to the fact that only 3 participants were scored by 3 raters, ICC (2,3) = 0.90, 95%CI [0.19 to 1.0], *F*(2,4) = 110.34, *p* < .001, reflecting a poor to excellent IRA. The IRA for the B scale can be considered as good to excellent, ICC (2,3) = 0.98, 95%CI [0.82 to 1.00], *F*(2,4) = 65.60, *p* < .01. This indicates that the raters had a high agreement in their scores for the B scale, meaning that they often assigned exactly the same scores to the same items.

### Validity

#### Criterion Validity

To determine optimal cut-off scores for the Dutch version of the 3Di-Adult, ROC-curves were computed per A and B scale, using the diagnosis made by an independent clinician as the criterion. For the A scale, the AUC was 0.93, 95%CI [0.88-0.98], *p* < .001, reflecting high accuracy. The highest Youden’s *J*, i.e. *J =* 0.74, for the A scale was found for the cut-off score of 1.06, resulting in a sensitivity of 83.9% and a specificity of 90%. For the B scale, the AUC was 0.95, 95%CI[0.91-0.99], *p* < .001, reflecting high accuracy. The highest Youden’s *J*, i.e. *J* = 0.74, was found for a cut-off score of 1.37 for the B scale, resulting in a sensitivity of 94% and a specificity of 80%.

Hence, we calculated the number of participants being correctly classified as ASD by the 3Di-Adult – i.e. a score of ≥ 1.06 on the A scale *and* a score of ≥ 1.37 on the B scale – and as control participant – i.e. a score of < 1.06 on the A scale and/or a score of < 1.37 on the B scale (see Table 3), when compared to their clinical diagnosis (i.e. the criterion). A total of 50 out of 62 ASD participants got correctly classified as ASD, i.e. sensitivity of 80.6%. Of the 12 false negative cases, 2 participants scored below the cut-off score of both the A and the B scale, whereas 8 participants scored below the cut-off on the A scale only, and 2 participants scored below the cut-off of the B scale only. The AQ-Short total scores of all ASD participants were above the suggested cut-off score for an ASD diagnosis of 65.

**Table 3:** Agreement between clinical diagnosis and 3Di-Adult diagnosis using cut-off scores as determined by ROC-curves

		Golden standard		Total (n = 92)
		ASD (n = 62)	Control (n = 30)	
Scale A	ASD range^a^	52	3	55
	Control range	10	27	37
Scale B	ASD range^b^	58	6	64
	Control range	4	24	28
A and B	ASD range	50^c^	2^d^	52
	Control range	12^e^	28^f^	40

Note. In order to be classified as ASD according to the 3Di-Adult, a score above the cut-off of the A *and* the B scale was required

^a^ Referring to a score of ≥ 1.06

^b^ Referring to a score of ≥ 1.37

^c^ True positive cases

^d^ False positive cases

^e^ False negative cases

^f^ True negative cases

A total of 28 of 30 control participants were correctly classified as control participants, i.e. specificity of 93.3%. The remaining two false positive cases scored above the cut-off on both the A and the B scale. Their AQ-Short total scores were 51 and 58, which were below the cut-off of 65 for ASD. Of the 28 true negative cases, 1 participant scored above the cut-off score on the A scale and 4 participants scored above the cut-off score of the B scale.

The positive predictive value of the 3Di-Adult was 96.2%, referring to the percentage of participants that scored above the threshold of the 3Di-Adult – i.e. above the cut-off score on the A *and* B scale – that actually had ASD according to their clinical diagnosis. The negative predictive value was 70.0%, reflecting the percentage of participants that scored below the threshold and were control participants (i.e. no diagnosis of ASD).

#### Construct Validity

##### Convergent Validity: 3Di-Adult and the AQ-Short

For investigation of the convergent validity, correlations between the 3Di-Adult and the AQ-Short were calculated. The 3Di-Adult A scale was expected to be significantly correlated with the AQ-Short higher order scale of ‘social behavioral difficulties’ and the 3Di-Adult B scale was expected to be significantly correlated with the AQ-Short scale of ‘a fascination with numbers and patterns’. For the total sample, the A scale correlated moderately, significantly with the AQ-Short ‘social behavioral difficulties’ scale (*r*_*s*_ = 0.60, *p* < .001). The B scale correlated moderately, significantly with the AQ-Short scale ‘a fascination with numbers and patterns’ (*r*_*s*_ = 0.57, *p* < .001). Correlations per ASD and control group showed insignificant correlations between the A scale and the AQ-Short scale ‘social behavioral difficulties’ (*r*_s_’s < 0.36), and the B scale and the AQ-Short scale ‘a fascination with numbers and patterns’ (*r*_s_’s < .29).

##### Divergent Validity: 3Di-Adult and Demographic Measures

*Participants’ Characteristics* An independent samples t-test revealed that males had significantly higher scores on the A scale (mean = 1.96, *SD* = 1.09) compared to females (mean = 1.33, *SD* = 0.97), for the total sample, *t*(87) = 2.82, *p* = .006. However, when the analysis was run per ASD and control group, significance was only found in the ASD group (ASD males: mean = 2.30, *SD* = 0.88, ASD females: mean = 1.83, *SD* = 0.83) *t*(59) = 2.11, *p* = .04), but not in the control group (males: mean = 0.56, *SD* = 0.65; females: mean = 0.50, *SD* = 0.49) *t*(26) = 0.23, *p* = .82). On the B scale, a significant difference was found for the total sample in which males scored significantly higher (mean = 3.02, *SD* = 1.61) than females (mean = 2.29, *SD* = 1,67), *t*(87) = 2.01, *p* < .05. However, when analyzing these differences per group, no significant differences were found (ASD group; mean_males_ = 3.56, *SD*_males_ = 1.25, mean_females_ = 3.24, *SD*_females_ = 1.31, *t*(59) = 0.96 *p* = .34, control group; mean_males_ = 0.82, *SD*_males_ = 0.92, mean_females_ = 0.74, *SD*_females_ = 0.83 *t*(26) = 0.20, *p* = .85).

Spearman’s rho revealed no significant correlation between age and the A scale (*r*_*s*_ = − 0.16, *p* = .12) nor with the B scale (*r*_*s*_ = − 0.15, *p* = .63) for the total sample. Analyses within groups revealed a bordering significance trend for the ASD group with somewhat lower scores for older ages (A scale: *r*_*s*_ = -0.25, *p* = .06, B scale: *r*_*s*_ = -0.22, *p* = .09), which was not found in the control group (A scale: *r*_*s*_ = 0.06 *p* = 75, B scale: *r*_*s*_ = 0.10, *p* = .63). An ANOVA showed that education levels of the participant – divided in three levels – was not related to the A scale (*p* = .28) nor the B scale (*p* = .24) for the total sample, nor for the ASD and control group separately (*p*’s > 0.77). Intelligence level of the participants – divided in 3 levels - was not related to both the A scale (*p* = .36) nor the B scale (*p* = .28) for the total sample, nor for the ASD group and control group separately (*p’s* > 0.31).

*Informants’ Characteristics* Age of the informant was not correlated with the A scale (*r*_*s*_ = − 0.07, *p* = .49) and B scale (*r*_*s*_ = − 0.11, *p* = .31) for the total sample, nor within the ASD and control group (*r*_*s*_*‘s* <-.18, *p*’s > 0.16). No significant differences were found between gender of the informant and scores on the A scale (*t*(89) = -1.38, *p* = .17) and B scale (*t*(89) = -0.80, *p* = .43) for the total sample, nor within the ASD and control group (*p*’s > .31). An ANOVA revealed that education level of the informant was not related to the A scale (*p* = .89), nor the B scale (*p* = .52) for the total sample, nor for within the ASD and control group separately (*p’s* > .24).

## Discussion

In the current study, we evaluated the psychometric properties of the Dutch 3Di-Adult, an informant-based, structured interview intended to assess ASD in adults based on DSM-5 criteria. Results indicate that the Dutch 3Di-Adult is a valid, reliable and time-efficient tool to assess ASD in adults in less than 45 min. The 3Di-Adult showed high internal consistency measures, as well as promising inter-rater agreement and inter-rater reliability measures. Furthermore, the 3Di-Adult showed a good ability to discriminate ASD participants from non-ASD participants, as reflected by high sensitivity and specificity measures. Our findings suggest a moderate convergent validity with moderate, significant correlations between the 3Di-Adult and the AQ-Short for the total sample. Tests with respect to the divergent validity showed that males scored significantly higher, i.e. more autistic features, than females on both the A and the B scale of the 3Di-Adult for the total sample. No significant relationships were found between the 3Di-Adult and participants’ and informants’ education level, intelligence level, and age though a bordering significant trend was found for less autistic features in older ASD participants compared to younger ASD participants.

The average administration time was around 41 min, which makes the 3Di-Adult a substantially faster tool than the ADI-R or the DISCO, which can take up to three hours (Lord et al., 1994; Wing et al., 2002). The 3Di-Adult took longer for the ASD group (mean = 46.55 min) than for the control group (mean = 31.93 min). In the current study, this difference in duration was probably mostly seen, because informants in the ASD group recognized the asked behaviors and elaborated on these by giving rich examples. The administration time was not related to the number of missing items during the interview – i.e. the number of questions the informant answered ‘don’t know’ to. The number of missing items was not related to group (ASD versus control), indicating that informants of both groups were equally able to answer the questions asked.

The 3Di-Adult showed high internal consistency for both the A and the B scale. Additionally, the inter-rater reliability was good to excellent, indicating that raters consistently discriminated between different items. Inter-rater agreement was difficult to interpret for the A scale due to the broad 95% confidence interval, which may be a result of the inclusion of only three participants. Agreement was good to excellent for the B scale, meaning that raters often assigned exact same scores to the same items for the B scale.

The Dutch 3Di-Adult shows good sensitivity (80.6%) and specificity (93.3%), indicating that the participants with ASD were in 80.6% percent of the cases correctly classified as ASD by the 3Di-Adult, and that control participants were correctly classified as non-ASD by the 3Di-Adult in 93.3% of the cases. Furthermore, even though there are no official guidelines for interpreting the percentages of the PPV and NPV, the PPV of the current study seems to be high (i.e. 96.2%), indicating that most of the participants that were classified as ASD by the 3Di-Adult, indeed had ASD according to the gold standard, i.e. a diagnosis obtained by an independent clinician. The NPV seemed adequate (70.0%), indicating that 70% of the participants that were classified as non-ASD by the 3Di-Adult, were indeed control participants – i.e. no ASD diagnosis. However, it is important to note that the PPV and NPV values are highly dependent on the prevalence of ASD in the sample being investigated (Tenny & Hoffman, 2020). Samples with a higher prevalence of the condition being investigated – in this case ASD – tend to produce higher PPV values and lower NPV values as it is simply ‘easier’ to find an ASD case. Therefore, future research should test the 3Di-Adult in a sample in which the prevalence of ASD is close to the prevalence of the setting in which the instrument will be used.

Our results are in line with expectations and previous research by Mandy and colleagues (2018), who found good reliability and validity for the English version of the 3Di-Adult. Mandy and colleagues reported a higher sensitivity (i.e. 95%) and slightly lower specificity (i.e. 92%) than was found in the current study among Dutch participants (i.e. 80.6% and 93.3%). This may be due to natural fluctuation or random error, but it may also be a result from differences between the samples. In the study of Mandy and colleagues, scores on the A and B scale showed larger differences between the ASD group and control group compared to our study. Therefore, it may have been slightly more challenging to discriminate the ASD group from the control group in the current sample. In addition, the sample of Mandy and colleagues included a broader range of intelligence levels, and contained more males, whereas the current sample consisted of more females and higher intelligence levels. Females with ASD tend to show less obviously autistic social behavior compared to males with ASD (Kopp & Gillberg, 2011). Autistic females with normal range IQ are known to use camouflaging strategies, which could make it more difficult to diagnose their autistic behavior (Bargiela et al., 2016). In the current (total) sample, we found that females scored significantly lower on the 3Di-Adult than males, i.e. displaying less autistic features. However, when associations were calculated for the ASD and control group separately, ASD females scored lower on the A scale but not on the B scale. Based on our results, we advise to consider creating cut-off scores for men and women separately. Future research, including larger and more representative sample sizes should further investigate this.

Convergent validity was assessed by calculating the correlations between the 3Di-Adult and the AQ-Short. Moderate, significant correlations were shown for the total sample, indicating moderate convergent validity. However, when correlations were calculated per group (ASD versus control), the correlations became insignificant. Though this may be a result of less power due to lower sample sizes, it may also reflect the complementing information both instruments provide. As all ASD participants obtained a score above the cut-off for ASD on the AQ-Short, and most ASD participants also scored above the cut-off for ASD on the 3Di-Adult, both instruments seem to provide relevant, complementing, information regarding ASD features. We believe that this highlights the need for a multi-informant approach to create a complete picture of the experience of the individual and the environment as they may provide different information (Rankin et al., 2017). The need for a multi-informant approach was also emphasized by our results of the self-report AQ-Short total scores of the participants that were incorrectly identified by the 3Di-Adult (i.e. false negative and false positive cases). Their AQ-Short total score did show agreement with their group status (i.e. ASD or control), providing important additional information to the 3Di-Adult results.

Results regarding the divergent validity showed that - aside from males scoring significantly higher, i.e. more autistic, than females in the total sample – no relationship was found between scores on the 3Di-Adult and age, education level and IQ levels of both the participant and the informant. However, a trend was found within the ASD group for age and scores on the 3Di-Adult, in which the scores seemed to decrease when age increased. This is in line with literature showing that autistic features seem to become less visible when people age (Charman et al., 2011; Shattuck et al., 2007), which may be due to various reasons such as well-acquired camouflaging strategies. Potentially, cut-off scores for the 3Di-Adult should be adjusted to the age of the participant, but more research is needed to draw firm conclusions.

Several limitations to the study have been identified. First, using a sample consisting of 2 clearly different and pre-distinguished groups - i.e. participants with ASD diagnosis versus without an ASD diagnosis – might have made it relatively easy for the interview to distinguish the groups. In clinical practice, adults being tested are not in one of these clear groups (yet) and may show subclinical autistic features, setting a more difficult task for the interview(er). Therefore, the current sensitivity and specificity may be overestimated. However, it was not entirely certain that our control participants did not have autism, as the requirement was to have no official ASD diagnosis but not to have had an ASD assessment to rule out autism. Potentially, some of our control participants were willing to participate in the study out of interest in autism because they recognized some of their own behaviors as being somewhere along the autism spectrum. Initially, we considered this to be a limitation of the study, but in light of the first limitation, it may have led to less inflated sensitivity and specificity measures. Another limitation is that our sample was not representative, as ASD seems to be more prevalent among males and in people with a below average intelligence level (Charman et al., 2011; Loomes et al., 2017), though the prevalence numbers may be biased, because ASD might be more visible (better observable), and therefore easier to diagnose, in males and amongst people with a below average intelligence level (Dean et al., 2017; Höfer et al., 2019). Another shortcoming, which was also present in the study of Mandy and colleagues, was that the interviewers and informants were not blind to condition. This may have influenced the behavior of both parties: the interviewer may have had certain expectations, resulting in a confirmation bias. Informants of participants with ASD are likely to already have experience with these type of questions, and have acquired knowledge about the autistic behavior of the participant, enabling them to more easily report about the autistic traits compared to when the informant would not know whether the participant has ASD (e.g. when the participant is still in a diagnostic procedure). However, there were also multiple strengths to the study. All raters received the same official, certified 3Di-Adult training. In addition, in order to assess the inter-rater reliability and inter-rater agreement, the second and third rater were blind to the condition of the participant (i.e. ASD or control). Furthermore, a multi-informant approach was used, i.e. self-report and informant-report measures, which provides more reliable information compared to single-informant procedures.

Further research should assess the psychometric properties of the 3Di-Adult in a more representative sample with raters and participants blind to their autism diagnostic status. This can be achieved by administering the interview to consecutive referrals presenting to diagnostic services, prior to the outcome of their autism assessment. This is of high importance, since a design in which the participants already obtained a diagnosis is likely to produce an inflated sensitivity. It will also be useful to design studies incorporating a clinical comparison group to investigate whether the 3Di-Adult is able to discriminate between ASD and other psychiatric conditions that may overlap with autistic symptomatology. Furthermore, it would be interesting to carry out a subgroup analysis on type of informant. In this study, informants were in most cases the mother. However, it should be sorted out whether information provided by a sibling, or even a partner, is as reliable as the information given by the (often) primary caregiver. Last, as we found a trend in the ASD group for lower 3Di-Adult scores with increasing age, more research regarding the impact of age on 3Di-Adult scores is needed, including reasons for why this is the case (i.e. acquired camouflaging strategies) using a sample with a broader and preferably evenly distributed age range.

Results of the current study seem promising and allow us to formulate certain implications and clinical recommendations. The 3Di-Adult can be a useful tool contributing to the diagnostic process of adults with suspected (or unsuspected) ASD. We emphasize that the 3Di-Adult should not be used alone to make clinical diagnostic decisions, but rather should form one component of a multi-modal assessment in which direct observation, self-report and informant report are included (National Institute for Health and Care Excellence, 2016). Moreover, it provides the clinician with a broader view of the participant, including strengths (e.g. finds it easy to meet new people) of the person but also challenges that are encountered (e.g. difficulties with maintaining relationships), making it a useful tool for further guidance. Furthermore, considering the relatively fast administration time, the 3Di-Adult may be a helpful tool for researchers as well because they often want to confirm their participants’ (self-reported) diagnosis, while using the ADI-R or the DISCO – other informant-based interviews - have a much longer administration time (De Bildt et al., 2004; Lord et al., 1994; Wing et al., 2002). Since the 3Di-Adult is a substantially faster interview with promising reliability and validity measures, it may form a valuable alternative for the currently used interviews. Further research is needed to test this assumption.

## Data Availability

Data can be shared based upon reasonable request.
